# Murine startle mutant *Nmf11* affects the structural stability of the glycine receptor and increases deactivation

**DOI:** 10.1113/JP272122

**Published:** 2016-05-10

**Authors:** Megan E. Wilkins, Alex Caley, Marc C. Gielen, Robert J. Harvey, Trevor G. Smart

**Affiliations:** ^1^ Department of Neuroscience, Physiology and PharmacologyUniversity College LondonGower StreetLondonWC1E 6BTUK; ^2^ Department of PharmacologyUCL School of Pharmacy29–39Brunswick SquareLondonWC1N 1AXUK

## Abstract

**Key points:**

- Hyperekplexia or startle disease is a serious neurological condition affecting newborn children and usually involves dysfunctional glycinergic neurotransmission.
- Glycine receptors (GlyRs) are major mediators of inhibition in the spinal cord and brainstem.
- A missense mutation, replacing asparagine (N) with lysine (K), at position 46 in the GlyR α1 subunit induced hyperekplexia following a reduction in the potency of the transmitter glycine; this resulted from a rapid deactivation of the agonist current at mutant GlyRs.
- These effects of N46K were rescued by mutating a juxtaposed residue, N61 on binding Loop D, suggesting these two asparagines may interact.
- Asparagine 46 is considered to be important for the structural stability of the subunit interface and glycine binding site, and its mutation represents a new mechanism by which GlyR dysfunction induces startle disease.

**Abstract:**

Dysfunctional glycinergic inhibitory transmission underlies the debilitating neurological condition, hyperekplexia, which is characterised by exaggerated startle reflexes, muscle hypertonia and apnoea. Here we investigated the N46K missense mutation in the GlyR α1 subunit gene found in the ethylnitrosourea (ENU) murine mutant, *Nmf11*, which causes reduced body size, evoked tremor, seizures, muscle stiffness, and morbidity by postnatal day 21. Introducing the N46K mutation into recombinant GlyR α1 homomeric receptors, expressed in HEK cells, reduced the potencies of glycine, β‐alanine and taurine by 9‐, 6‐ and 3‐fold respectively, and that of the competitive antagonist strychnine by 15‐fold. Replacing N46 with hydrophobic, charged or polar residues revealed that the amide moiety of asparagine was crucial for GlyR activation. Co‐mutating N61, located on a neighbouring β loop to N46, rescued the wild‐type phenotype depending on the amino acid charge. Single‐channel recording identified that burst length for the N46K mutant was reduced and fast agonist application revealed faster glycine deactivation times for the N46K mutant compared with the WT receptor. Overall, these data are consistent with N46 ensuring correct alignment of the α1 subunit interface by interaction with juxtaposed residues to preserve the structural integrity of the glycine binding site. This represents a new mechanism by which GlyR dysfunction induces startle disease.

## Key points

- Hyperekplexia or startle disease is a serious neurological condition affecting newborn children and usually involves dysfunctional glycinergic neurotransmission.
- Glycine receptors (GlyRs) are major mediators of inhibition in the spinal cord and brainstem.
- A missense mutation, replacing asparagine (N) with lysine (K), at position 46 in the GlyR α1 subunit induced hyperekplexia following a reduction in the potency of the transmitter glycine; this resulted from a rapid deactivation of the agonist current at mutant GlyRs.
- These effects of N46K were rescued by mutating a juxtaposed residue, N61 on binding Loop D, suggesting these two asparagines may interact.
- Asparagine 46 is considered to be important for the structural stability of the subunit interface and glycine binding site, and its mutation represents a new mechanism by which GlyR dysfunction induces startle disease.

**Abbreviations**
DMEM: Dulbecco's modified Eagle medium
DR: dose‐ratio
ENU: ethylnitrosourea
GFP: green fluorescent protein
*GLRA1*: GlyR α1 subunit gene
*GLRB*: GlyR β subunit gene
GluCl: glutamate‐activated Cl^˗^ channel
GlyR: glycine receptor
HEK‐293: human embryonic kidney 293 cells
K_B_: equilibrium dissociation constant
V_patch_: trans‐patch potential
WT: wild type

## Introduction

Hyperekplexia or startle disease is a serious neurological condition affecting newborn children. It is characterised by exaggerated startle reflexes following tactile and acoustic stimuli, resulting in hypertonia and apnoea. Although considered as a rare orphan disease (<200,000 affected individuals world‐wide), this disorder can cause developmental delay and sudden infant death (Davies *et al*. 2010). The underlying cause of hyperekplexia involves dysfunctional glycinergic transmission (Harvey *et al*. 2008) and causative mutations are typically found in the genes encoding GlyR α1 (*GLRA1*; Shiang *et al*. 1993, 1995; Chung *et al*. 2010) and β subunits (*GLRB*; Rees *et al*. 2002), and the presynaptic glycine transporter, GlyT2 (Rees *et al*. 2006).

Animal models of startle disease are crucial for understanding the complex genetics of hyperekplexia and characteristic symptoms are exhibited by several mouse mutants harbouring different mutations in the GlyR α1 subunit gene (*GLRA1*), including: *spasmodic*, *oscillator*, *cincinatti* (Graham *et al*. 2006; Schaefer *et al*. 2013) and *Nmf11* (Traka *et al*. 2006). In the *spasmodic* mouse, a missense mutation (A52S) in the extracellular domain of GlyR α1, caused a relatively mild phenotype, with homozygous mice appearing normal at rest but developing an exaggerated startle response to acoustic or tactile stimuli at around 2 weeks of age (Lane *et al*. 1987). Although located outside the ligand‐binding domain, A52S reduced the sensitivity to glycine and the co‐operativity of binding with increased ligand occupancy (Ryan *et al*. 1994; Plested *et al*. 2007). By contrast, *oscillator* homozygotes and the spontaneous mutant *cincinatti* exhibit a more severe lethal phenotype due to a microdeletion in *Glra1* exon 8 or duplication of *Glra1* exon 5, respectively, causing a complete loss of functional GlyRs (Kling *et al*. 1997; Graham *et al*. 2006). The ENU‐induced mutant, *Nmf*11, also produces a lethal phenotype following a missense mutation (N46K) in the extracellular domain of GlyR α1 (Traka *et al*. 2006). The lethality of the *Nmf11* mutation (N46K) is, however, puzzling, because neither α1 subunit protein levels nor the somatodendritic distribution of GlyRs are affected, discounting trafficking or clustering deficits (Traka *et al*. 2006).

Although N46 lies in proximity to the glycine binding site, it does not form part of an identified binding loop or transduction pathway. However, from homology modelling and from glycine receptor structures at atomic level resolution, N46 is located at the subunit–subunit interface, opposing binding loop A (Vafa *et al*. 1999; Du *et al*. 2015; Huang *et al*. 2015) and sited between loops D and F, which are involved in agonist binding (Miller & Smart, 2010). We found that the GlyR α1 sensitivity for glycine was substantially reduced by N46K due to an increased rate of glycine deactivation of the mutant receptor. Our data identify an approximate threshold for the reduction in glycine potency that results in lethality of GlyR mutant mice, in addition to uncovering a role for N46 in GlyR activation/ deactivation and a new mechanism for hyperekplexia.

## Methods

### HEK cell transfection and mutagenesis

Human embryonic kidney (HEK293) cells were grown in DMEM supplemented with 10 % v/v fetal calf serum, 2 mm l‐glutamine, 100 u ml^−1^ penicillin‐G and 100 μg ml^−1^ streptomycin in a 5 % CO_2_–air atmosphere at 37 °C. Cells were harvested and plated onto poly‐l‐lysine‐coated 22 mm coverslips (VWR, Leuven, Belgium) and transfected with recombinant murine GlyRs 1–3 h later using a modified calcium phosphate precipitation method. For homomeric GlyRα1, the cells were co‐transfected with 1.5 μg pcDNA3‐ wild‐type or mutant rGlyR α1 with 1.5 μg pRK5‐eGFP for identifying transfected cells (1:1 ratio). For heteromeric αβ GlyRs, cells were co‐transfected with pcDNA3‐rGlyR β subunit (α:β:GFP in a 1:10:1 ratio; 4 μg total DNA). Mutations were made using the Stratagene Quikchange method. To increase efficiency, the rGlyRα1 N61 mutation PCR products were subject to *DpnI* digestion (NEB, Hitchin, UK), and methylation (NEB) prior to ligation. All mutated cDNAs were completely sequenced (Source Biosciences, Nottingham, UK) to check the inclusion and position of the mutations.

DNA solutions were incubated with 20 μl of 340 mm CaCl_2_ and 24 μl of double‐strength Hanks’ balanced salt solution (280 mm NaCl; 2.8 mm Na_2_HPO_4_; 50 mm Hepes; pH 7.2) for 5–10 min prior to drop‐wise addition to the plated cells. After 16–48 h post transfection, cells were used for electrophysiological recording.

### Whole‐cell recording

Glycine‐activated currents were recorded from single transfected HEK293 cells using whole‐cell patch clamp recording with an Axopatch 200B amplifier. Patch electrodes (resistances of 3–4 MΩ) were filled an internal solution containing (mm): 120 KCl, 1 MgCl_2_, 1 CaCl_2_, 10 Hepes, 11 EGTA, 30 KOH, 2 ATP and 0.5 GTP, pH 7.11 with 1 m NaOH. The cells were continuously perfused with a physiological salt solution containing (mm): 140 NaCl, 4.7 KCl, 1.2 MgCl_2_, 2.5 CaCl_2_, 5 Hepes, and 11 d‐glucose, pH 7.4.

HEK293 cells were voltage‐clamped at −10 mV and visualised using a Nikon Diaphot 300 microscope configured for differential interference contrast and epifluorescence. A Y‐tube enabled the rapid application of drugs to the HEK cells. Data were recorded directly to a Dell Pentium 4 computer via a Digidata 1320A (Molecular Devices, Sunnyvale, CA, USA) sampling at 15 kHz and filtered at 5 kHz (6th order Bessel). The currents were normalised to the maximum response amplitude activated by a saturating glycine concentration applied to each cell. Maximal responses, half‐maximal concentrations (EC_50_) and Hill coefficients were determined from concentration–response data fitted using the Hill equation with non‐linear least squares routines (Origin 6.0) as previously described (Miller *et al*. 2005 *a*). The biphasic curve data that resulted from the modulation of GlyR function by Zn^2+^ was fitted using a modified Hill equation as previously described (Miller *et al*. 2004). Any change that exceeded 10% of the membrane conductance and/or series resistance resulted in cessation of the recording. For all recordings, series resistance compensation to ∼80% was achieved.

### Schild analysis

Glycine concentration–response curves were constructed in the absence and presence of three different concentrations of strychnine. From the parallel curve shifts, the glycine concentrations that produced 50% of the maximum response in the absence (*d*) and presence (*d*_s_) of strychnine were measured. The dose‐ratios (DR = *d*_s_/*d*) were calculated and used in the Schild plot of log (DR − 1) against the log antagonist concentration ([B]) (Arunlakshana & Schild, 1959). Initially, the unweighted data were fitted with a power function of the form:

$$\mathrm{DR}-1=c{\left[B\right]}^{n},$$

where *c* is a constant and *n* is the slope of the plot. In all analyses, the slopes of the lines were not significantly different from unity (*P* > 0.05, two‐tailed *t* test) as expected for an antagonist that acts in a purely competitive manner. The data were then re‐fitted with the Schild equation using a constrained slope of 1:

$$\left(\mathrm{DR}-1\right)=\left[B\right]/K{\;}_{B}.$$

The intercept of the line when (DR − 1) = 1 enabled the equilibrium constant for strychnine (*K*_B_) to be determined. The upper and lower 95% confidence limits of the regression line were also calculated.

### Single‐channel recording analysis

Single glycine‐activated channel currents were recorded from cell‐attached patches from transfected HEK293 cells expressing WT and N46K homomeric α1 subunit GlyRs. Patches were held at +100 mV and the recorded currents were filtered at 5 kHz and sampled at 30 kHz. Thick‐walled borosilicate patch electrodes (8–10 MΩ) were filled with external physiological salt solution containing (mm): 102.7, NaCl; 20, sodium gluconate; 4.7, KCl; 2, CaCl_2_.2H_2_O; 1.2, MgCl_2_.6H_2_O; 10, Hepes; 14, glucose; 15, sucrose; 20, TEACl; pH 7.4; 320 mosmol l^–1^. For single‐channel experiments with WT GlyRs, the pipette solution incorporated an EC_60_ concentration of either glycine (0.05 mm) or taurine (0.4 mm). For the N46K experiments, the pipette solution was supplemented with equivalent EC_60_ concentrations of either glycine (0.5 mm) or taurine (0.9 mm).

Stored, pre‐filtered single‐channel records were assessed for simultaneous opening of multiple channels, but this usually formed less than 5% of the total opening events. These multiple openings were not used in the analyses. Currents were analysed using Strathclyde electrophysiology software (John Dempster, WinEDR ver 3.5.2). Open and shut durations were measured using a 50% threshold cursor applied to the main single‐channel current amplitude in each patch. As with all threshold cursor methods for detecting single‐channel state transitions, very brief shuttings can be missed, increasing the duration of adjacent open periods (Mortensen & Smart, 2007), but with the large glycine single‐channel currents and their lack of sub‐conductance states, this was considered not to be a confounding problem. Dwell time frequency distributions were constructed from the detected individual open and shut durations. The minimum duration of resolvable events was set to 30 μs before fitting the dwell‐time histograms with one or more exponentials, defined by the equation below.

yt=∑i=1nAiτi exp −t/τ,

where *A*_i_ represents the area of the *i*th component to the distribution and τ_i_ represents the respective time constant. Using a Levenberg‐Marquardt non‐linear least‐squares routine, the areas of the individual exponential components, their relative time constants and standard errors of these parameters were determined. Clusters of channel activations were recognised by their separation from each other with long desensitised periods. Where this was not easily recognisable, we determined a critical shut time (τ_crit_; Colquhoun & Sakmann, 1985). This was determined between the longer shut time constants, τ_C2_ and τ_C3_, as described previously (Colquhoun & Sakmann, 1985; Mortensen *et al*. 2004; Mortensen & Smart, 2007). The number of transitions recorded per patch were 6000—12,000. Statistical significance was determined using an unpaired *t* test.

### Macropatch recordings

Outside‐out macropatches were voltage‐clamped at −20 mV and recorded currents filtered at 5 kHz and sampled at 30 kHz. Thick‐walled borosilicate patch electrodes (5–10 MΩ) contained the same internal solution used for whole‐cell recording. Cells were perfused with physiological salt solution. A theta glass electrode, pulled and cut with a diamond knife to a tip diameter of 50–100 μm, was used to apply adjacent solution streams for rapid exchange over the macropatch. Solution exchange was achieved by activating a piezoelectric transducer (Burleigh Instruments, Dortmund, Germany) that translated the solution interface for a specified time (2–200 ms). Open tip potentials were measured at the conclusion of each patch to confirm exchange rates (usually 150–300 μs). Application durations were programmed for multiple pulses, then averaged to reduce signal/noise ratios. Rise and deactivation times were calculated between 10 and 90 % of the peak current amplitude and reported as means ± SEM.

### Homology modelling

The sequence alignments between GlyRα1 and the *C. elegans* glutamate‐activated Cl^˗^ channel (GluCl; Hibbs & Gouaux, 2011) were constructed using ClustalW (Thompson *et al*. 1994). The mature GlyR α1 subunit was then modelled as a subunit interface dimer, based on the crystal structure template for GluCl (PDB 3RHW) in complex with Fab and ivermectin, using Modeller 9 ver. 7 (Sali & Blundell, 1993). The models with the lowest Discrete Optimised Protein Energy (DOPE) score were used and optimal side‐chain configurations were determined with SCWRL4 (Krivov *et al*. 2009). All structural images were rendered in PyMOL Molecular Graphics System (DeLano, Palo Alto, CA, USA; Pettersen *et al*. 2004). Subsequently, with the cryo‐electron microscopic and X‐ray crystallographic structures for zebrafish α1 (Du *et al*. 2015) and human α3 GlyRs (Huang *et al*. 2015) identified, we were able to refine our rGlyRα1 model using Modeller and PyMOL.

### Kinetic modelling

Channelab (ver 2, Synaptosoft, GA, USA) was used to generate the simulated whole‐cell currents to glycine on WT and N46K GlyRα1. The binding/unbinding rate constants, and gating and preactviation constants, are generally in accord with values previously determined and published for GlyRs (Burzomato *et al*. 2004). The desensitisation rates were empirically chosen to account for the profile of the current recordings for WT and N46K GlyRα1.

### Statistics

Statistical significance was determined using Graphpad Instat ver 3.06. Significant differences between groups of data were measured using an unpaired *t* test using raw or mean ± SEM data with *n* numbers stated in the text.

## Results

### N46K mutation reduces glycine potency

**Figure 1:**
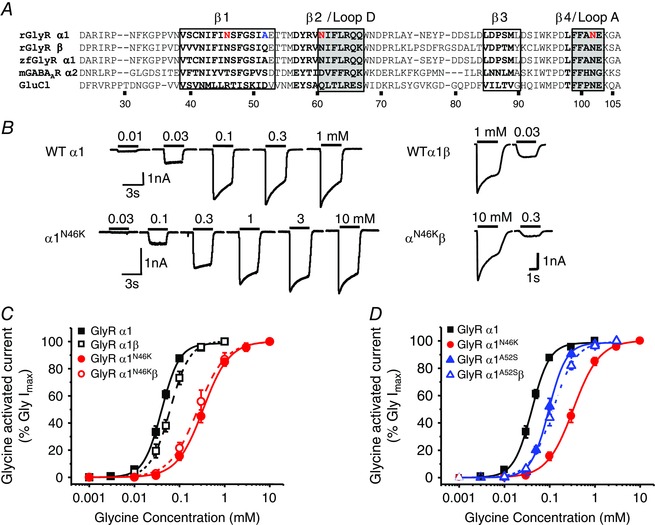
**N46K GlyR is less sensitive to glycine** *A*, primary sequence alignment comparing rat (r) and zebrafish (zf) GlyR α1 and β subunits to other pentameric ligand‐gated ion channel (pLGIC) family members: mouse (m) GABA_A_R α2 and *C. elegans* GluCl. The amino acid numbering refers to the mature rGlyR α1. The red highlighted residues indicate those mutated in this study. The black bold residues form β sheets, whilst those residues that form ligand binding loops and interspersed β loops are boxed. *B*, sample whole‐cell currents activated by 0.01, 0.03, 0.1, 0.3 and 1 mm glycine for WT α1 (upper left) and 0.03, 0.1, 0.3, 1, 3 and 10 mm glycine for α1^N46K^ (lower left). Right panels show EC_20_ and maximal glycine‐activated currents for HEK cells expressing heteromeric WT and N46K GlyRs. Bars indicate the durations of glycine application. *C*, concentration–response curves normalised to the maximal glycine‐activated response for homomeric α1 and heteromeric α1β wild‐type (WT) and N46K GlyRs. In all figures, the symbols represent means ± SEM. *D*, comparing concentration–response curves for glycine at WT, N46K and A52S mutant GlyRα1. The A52S homomeric and heteromeric GlyR EC_50_s of 101 ± 10 μm (*n* = 9) and 118 ± 4 μm (*n* = 6) are significantly lower than that for the N46K GlyR (*P* < 0.05).

Here we compared the glycine sensitivities of both homomeric and heteromeric GlyRs incorporating the N46K mutation in the α1 subunit with wild‐type receptors and the phenotypically less severe A52S mutation. Asparagine 46 is located in the extracellular domain, within the β1 loop (Brejc *et al*. 2002; Du *et al*. 2015) just before binding loop D of the GlyRα1 subunit (Fig. 1 *A*). Glycine‐activated currents for homomeric N46K GlyRα1 were comparable with those for WT receptors (Fig. 1 *B*) with saturating concentrations of glycine (1 and 10 mm, respectively) revealing only a small, but insignificant, decrease in maximal current: 3245 ± 570 pA (WT; *n* = 14) and 2209 ± 367 pA (N46K, *n* = 19, *P* > 0.05). However, the glycine concentration–response curves revealed differences in potency (Fig. 1 *C*). For GlyRα1 the glycine EC_50_ was 41 ± 3 μm (*n* = 20); whereas for GlyRα1^N46K^, glycine was 9‐fold less sensitive with an EC_50_ of 372 ± 4 μm (*n* = 19).

Heteromeric receptors (GlyRα1β) for WT and N46K exhibited comparable maximal glycine currents: 4840 ± 1019 pA (α1β, *n* = 6) and 4871 ± 460 pA (α1^N46K^β, *n* = 8, *P* > 0.05) with a smaller 5‐fold separation in receptor sensitivity to glycine; the respective EC_50_s being: 64 ± 6 μm (α1β; *n* = 6) and 318 ± 79 μm (α1^N46K^β; *n* = 8; Fig. 1 *C*). This suggested that the β subunit partly rescued the N46K hyperekplexia mutation effect on glycine current, which might be expected given that the β subunit is replacing mutant α subunits in the α1β heteromeric GlyR. Co‐assembly of α and β subunits was confirmed by a reduced inhibition of the EC_50_ glycine steady‐state response by picrotoxin (20 μm) (see below; Fig. 5 *F*). Thus, homomeric α1 GlyRs are the most sensitive to the N46K mutation.

As the phenotype for the *Nmf11* mouse was more severe (Traka *et al*. 2006) than that for *spasmodic*, which harbours the A52S mutation (Lane *et al*. 1987; Ryan *et al*. 1994), we compared the relative potencies of glycine. Glycine was more potent at α1^A52S^ than α1^N46K^ GlyRs, reflected by the relative displacements of the glycine concentration–response curves and the resulting glycine EC_50_s (Fig. 1 *D*).

### GlyR sensitivities to partial agonists and strychnine are affected by N46K

**Figure 2:**
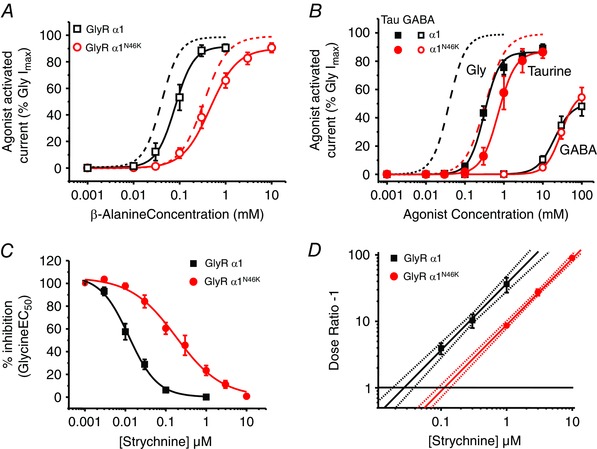
**N46K reduces partial agonist potency and strychnine affinity** *A*, β‐alanine concentration–response curves for WT and N46K GlyRs, normalised to the maximal glycine‐activated current for each cell. Glycine curves taken from Fig. 1 *C* are shown as dashed lines. The β‐alanine EC_50_s are: 90 ± 16 μm (WT; *n* = 7) and 552 ± 151 μm (N46K, *n* = 9) and relative maximum responses are WT = 92 ± 3%; N46K = 90 ± 3%. *B*, taurine and GABA concentration–response curves for activating homomeric WT and N46K GlyRs normalised to the maximum glycine‐activated current for each cell. Dashed lines are the curves for glycine activation of homomeric WT and N46K receptors from Fig. 1 *C* for comparison. *C*, inhibition concentration–response curves for strychnine antagonism of an EC_50_ glycine response for WT (IC_50_: 12.2 ± 2.2 nm; *n* = 8) and N46K (IC_50_: 193.4 ± 52.3 nm; *n* = 10). *D*, Schild plot analysis of competitive antagonism by strychnine of EC_50_ responses to glycine for WT and N46K GlyRs. Points represent mean dose‐ratios (DRs) from all cells examined at each antagonist concentration. The lines are fitted by linear regression and generated by the Schild equation with a constrained slope = 1. The dotted lines depict the upper and lower 95% confidence intervals. The intercepts with the line at DR ˗ 1 = 1, reveal the antagonist equilibrium constants.

To examine if the predominant effect of N46K is centred on the agonist binding site, the partial agonists β‐alanine, taurine and GABA were studied. For a full agonist, a reduction in efficacy can manifest as just a rightward displacement of the concentration–response curve; whereas a changed efficacy is often more evident with a partial agonist, revealed by an additional lower maximum response. β‐Alanine was 6‐fold less potent at N46K receptors compared to WT – without any change in the relative maximum response (Fig. 2 *A*). For the less potent partial agonists taurine and GABA, the curve displacements were less apparent: only 3‐fold for taurine (EC_50_: WT, 0.34 ± 0.03 mm; N46K 0.98 ± 0.23 mm; *n* = 7–8) and 1.5‐fold for GABA (EC_50_: WT, 21.27 ± 2.23 mm; N46K, 34.86 ± 6 mm; *n* = 5), again without any significant changes to the relative maximum responses (Fig. 2 *B*). This supported the hypothesis that N46K reduced ligand binding rather than agonist efficacy.

To further probe the role of N46K on the orthosteric binding site, inhibition by the competitive antagonist strychnine was examined (Ruiz‐Gomez *et al*. 1990; Vandenberg *et al*. 1992). The sensitivities of α1^WT^ and α1^N46K^ GlyRs to inhibition by strychnine were assessed with glycine EC_50_ responses. Strychnine concentration–inhibition curves indicated that the potency of the antagonist was reduced 15‐fold by N46K (Fig. 2 *C*), which is similar to the shift observed in glycine potency by N46K.

To determine if the reduced strychnine inhibition was due to a reduction in the binding affinity, we performed a Schild analysis. Shifts in the glycine concentration–response curve following exposure to 0.1, 0.3 and 1 μm strychnine for wild‐type, and 1, 3 and 10 μm for N46K were constructed. N46K caused an approximate 4.5‐fold increase in the strychnine equilibrium dissociation constant (*K*_B_) from 27.2 ± 4.21 nm (WT, *n* = 5) to 122.5 ± 16 nm (N46K *n* = 5; *P* = 0.0045; Fig. 2 *D*).

### Analysis of the N46 side‐chain

**Figure 3:**
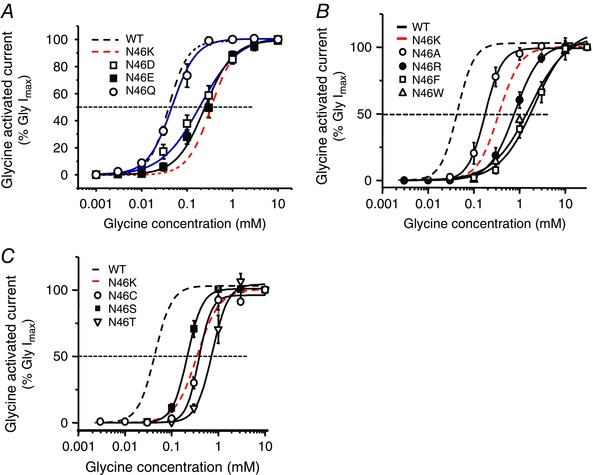
**Analysing the functional impact of residue 46 side‐chain** *A*, glycine concentration–response curves for GlyRs with conservative or charged N46 mutations. *B*, glycine concentration–response curves for mutations substituting N46 for alanine, phenylalanine, tryptophan or arginine. *C*, glycine concentration–response curves for N46 mutations with smaller polar side‐chain residues, lacking an amide group, including cysteine, serine and threonine. See Table 1 for EC_50_ values.

We analysed the importance of N46 by conservatively substituting it for glutamine (Q), which retains the amide side‐chain. The glycine concentration–response curve for N46Q (EC_50_ = 51 ± 4 μm; *n* = 5) was similar to that for wild‐type GlyRs (41 ± 3 μm; Fig. 3 *A*). However, concentration–response curves were displaced to the right when the amide side‐chain was replaced with the negatively‐charged carboxyl groups in aspartate and glutamate, N46D (202 ± 51 μm; *n* = 8) and N46E (272 ± 46 μm; *n* = 6; Fig. 3 *A*), and even more so with positively‐charged lysine (N46K, 372 ± 4 μm).

We next investigated whether side‐chain volume at position 46 had any impact on GlyR function. By substituting N46 for the smaller alanine, the glycine concentration–response curve was displaced to a lesser extent (A, EC_50_ 182 ± 26 μm, *n* = 5) compared with N46K. Replacing N46 with phenylalanine (F, EC_50_ 2.26 ± 0.57 mm; *n* = 6), a large non‐polar group, reduced the sensitivity of the GlyR even more than for N46K. Further increasing side‐chain volume with tryptophan (W; EC_50_ 1.45 ± 0.10 mm; *n* = 5), or changing charge using the basic amino acid arginine (R; EC_50_ 816 ± 130 μm; *n* = 6) did not cause any greater displacement in the concentration–response curve compared with N46F (Fig. 3 *B*). Smaller polar side‐chain residues, lacking an amide group, such as cysteine (C; EC_50_ 330 ± 16 μm; *n* = 6), serine (S; EC_50_ 221 ± 22 μm; *n* = 6) and threonine (T; EC_50_ 756 ± 133 μm; *n* = 6), again significantly reduced glycine potency compared with WT (Fig. 3 *C*; Table 1). These results demonstrate the critical importance of the polar amide side‐chain at position 46 for GlyR function.

**Table 1:** Glycine potency at N46 mutant GlyRs

**GlyRα1**	**EC_50_ (μm)**	**Comparison with WT (*P*)**	**Comparison with N46K (*P*)**	***n***
WT	41 ± 3	—	—	20
N46K	372 ± 4	< 0.0001	—	19
N46Q	51 ± 4	0.0766	< 0.0001	5
N46D	202 ± 51	0.0253	0.0208	6
N46E	272 ± 46	0.0041	0.082	6
N46A	182 ± 26	0.0057	0.0019	5
N46F	2260 ± 570	0.0115	0.0212	6
N46W	1450 ± 100	0.0001	0.0004	5
N46R	816 ± 130	0.0019	0.0189	6
N46C	330 ± 16	< 0.0001	0.048	6
N46S	221 ± 22	0.0005	0.001	6
N46T	756 ± 133	0.003	0.0343	6

EC_50_ values for WT and N46 mutant GlyRs are shown. All values are means ± SEM for *n* cells. *P* values are shown for the comparisons of the EC_50_ mean ± SEM values for mutant receptors with WT or N46K counterparts.

### The structural role of N46

**Figure 4:**
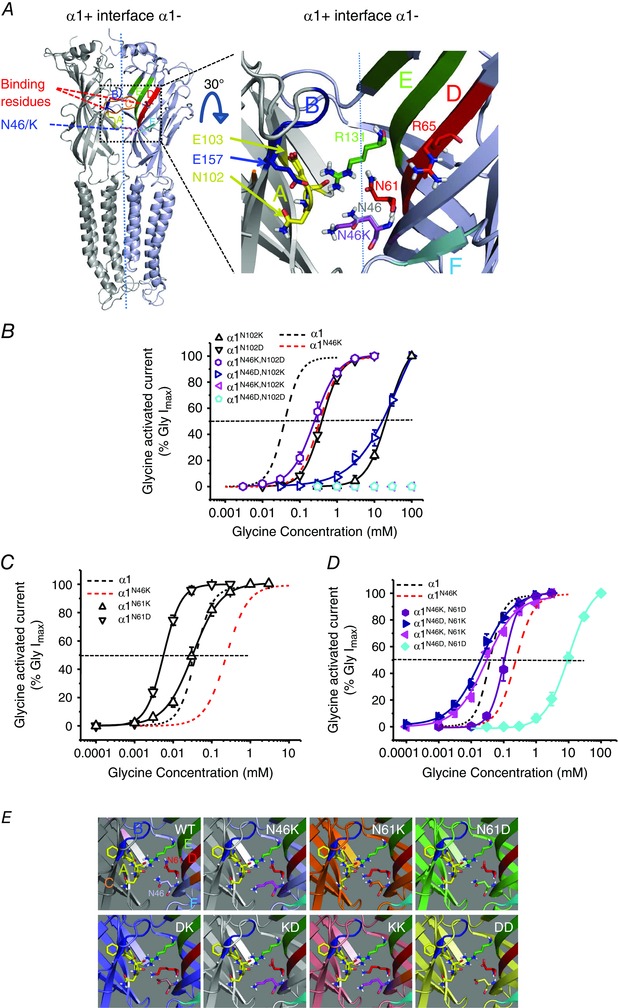
**Structure–function analyses of N46** *A*, left panel, homology model of two adjacent GlyR α1 subunits. Right panel, expanded 30^o^ tilted view of the α1 subunit–subunit interface. The locations for N46 (grey) and K46 (purple) are shown, as well as two key glycine binding residues: R65 (red, loop D) and E157 (blue, loop B); and relative side‐chain orientations for N61 (red) and R131 (green) at the complementary (˗) subunit interface, and N102/E103 (yellow) located on the principal side (+) of the interface. The glycine binding loops are: A (yellow), B (blue), C (orange; removed for clarity), D (red), E (green) and F (cyan). *B*, glycine concentration–response curves for reverse charge mutations at N102 and N46. Single mutations of N102 to lysine or aspartate shifted the glycine curves to the right. Note substitutions of N46 and N102 with reverse charges (N46K‐N102D: EC_50_ 0.29 ± 0.07 mm; *n* = 7, N46D‐N102K; EC_50_ 41 ± 9 mm; *n* = 6) did not restore WT GlyR sensitivity to glycine. Substitution of both N46 and N102 with the same charged residues abolished sensitivity to glycine. *C*, the glycine curve for N61K overlays the WT curve, whilst N61D caused a shift to the left. *D*, glycine concentration–response curves for paired N46 and N61 mutant GlyRs. Exchanging N46 and N61 with reverse charge mutants regained some (N46K‐N61D) or all (N46D‐N61K) of the sensitivity to glycine. Substitution of N46 and N61 with the same charge, either recovered (N46K‐N61K) or reduced (N46D‐N61D) the sensitivity to glycine compared to N46K GlyRs. *E*, homology models for N46 and N61 mutations in relation to the surrounding residues in the same plane. Binding loops that are involved in the orthosteric binding site of pLGICs are shown colour‐coded: loop A (yellow), loop B (blue), loop C (orange; removed for clarity), loop D (red), loop E (green) and loop F (cyan). DK – N46D, N61K; KD – N46K, N61D; KK – N46K, N61K; DD – N46D, N61D.

Using structural homology modelling, based on the glutamate‐activated Cl^˗^ channel (GluCl), and the atomic resolution structures for the human α3 (Huang *et al*. 2015) and zebrafish α1 GlyR subunits (Du *et al*. 2015), located the N46K mutation to within close proximity of the orthosteric binding pocket; however, it was considered unlikely to directly contribute to glycine binding (Fig. 4 *A*). Furthermore, it seems unlikely that N46 fulfils an α‐helical capping action or stabilisation of a β loop, as the structural projections placed this residue in the middle of a β loop (Wan & Milner‐White, 1999). However, the location of N46 suggests it might interact with residues in binding loop A of the neighbouring α1 subunit, or with other residues in the same α1 subunit (e.g. in loop D; Fig. 4 *A*). Our receptor structures suggested that the side‐chains for loop A residues, N102 and E103, are directed away from N46 implying that direct interactions are unlikely (Fig. 4 *A*), with N102 more likely to interact with its own backbone (Pal & Sankararamakrishnan, 2008). To investigate, N102 was exchanged for lysine (K; EC_50_ 23 ± 2 mm; *n* = 7) or aspartate (D; EC_50_ 0.42 ± 0.05 mm; *n* = 4) resulting in 500‐ and 10‐fold shifts, respectively, in the glycine concentration–response curves compared to WT (0.041 ± 0.003 mm; Fig. 4 *B*). Charge reversal at N46 (to K or D) and at N102 (to D or K) did not reverse the displacement of the glycine curves to that observed with WT (N46K‐N102D: EC_50_ 0.29 ± 0.07 mm; *n* = 7, N46D‐N102K; EC_50_ 41 ± 9 mm; *n* = 6), suggesting it was unlikely that N46 directly interacted with N102 (Fig. 4 *B*). In addition, mutating N46 and N102 to the same charged residue resulted in non‐functional glycine receptors.

Our structural models of GlyRα1 predicted that asparagine 61, located within the same subunit as N46, but on the juxtaposed loop D that houses a critical glycine binding site residue R65 (Grudzinska *et al*. 2005; Fig. 4 *A*), was a prime candidate for interaction. Mutating N61 to lysine had no significant effect on the glycine concentration–response curve compared to WT. However, mutating N61 to aspartate displaced the curve towards lower glycine concentrations (Fig 4 *C* and *E*: Table 2).

**Table 2:** Concentration–response curve data for N46 and N61 charge reversal mutants

**GlyRα1**	**EC_50_ (μm)**	**Hill slope**	***I*_max_ (pA)**	***n***
WT	41 ± 3	2.96 ± 0.40	3245 ± 570	20
N46K	372 ± 4*	1.89 ± 0.12*	2210 ± 367	19
N46D	202 ± 51*	1.20 ± 0.07*	1551 ± 126*	8
N61K	37 ± 7	1.39 ± 0.07*	2627 ± 716	7
N61D	6 ± 0.3*	2.27 ± 0.16	2893 ± 217	7
N46DN61K (DK)	22 ± 5*	1.03 ± 0.09*	964 ± 172*	6
N46KN61D (KD)	123 ± 18*	2.41 ± 0.15	3835 ± 931	7
N46KN61K (KK)	29 ± 7	0.98 ± 0.11*	1397 ± 206*	8
N46DN61D (DD)	10,863 ± 1,255*	1.29 ± 0.16*	6685 ± 1548	8

EC_50_, Hill slope and maximum current (*I*_max_) parameters for WT GlyRs and selected mutants at N46 and N61 for *n* cells. All values are means ± SEM. *Significant difference from the WT value; *P* < 0.05.

Interestingly, mutations that involved paired charge reversals at N61 and N46 suggested these two residues might interact; glycine potency was either partially rescued by N46K‐N61D or completely rescued by the N46D‐N61K pairing (Fig. 4 *D* and *E*). The double mutant N46K‐N61K also rescued glycine potency back to WT levels, whilst N46D‐N61D significantly reduced glycine potency when compared with curves for WT and also N46K GlyRs (Fig. 4 *D* and *E*; Table 2).

### Allosteric modulation by Zn^2+^ and neurosteroids

**Figure 5:**
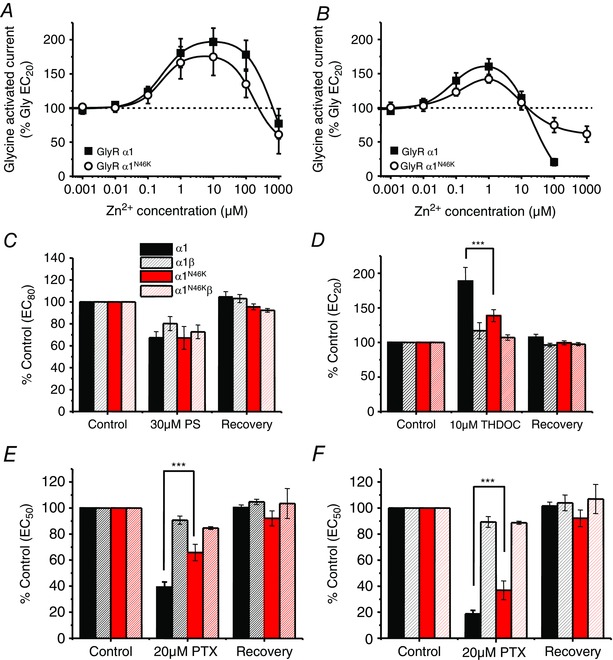
**Allosteric modulation at WT and N46K GlyRs** *A*, Zn^2+^ concentration modulation curves for the glycine EC_20_ response on WT and N46K GlyRα1. By co‐applying Zn^2+^ with EC_20_ glycine, there was no significant change in the potentiation or inhibition of the glycine‐activated peak current on WT compared with N46K GlyRα1. *B*, modulation of glycine EC_20_ responses following pre‐ and co‐application of Zn^2+^ (*n* = 5). *C*, effect of the neurosteroid, pregnenolone sulphate (PS; 30 μm; *n* = 5–9) on peak glycine EC_80_ currents for α1, α1β, α1^N46K^ and α1^N46K^β GlyRs. *D*, effect of the neurosteroid THDOC (10 μm; *n* = 4–11) on peak glycine EC_20_ currents for α1, α1β, α1^N46K^ and α1^N46K^β GlyRs. *E*, effect of picrotoxin (20 μm), a GlyR channel blocker, on peak EC_50_ glycine‐activated currents for α1, α1β, α1^N46K^ and α1^N46K^β GlyRs (*n* = 4–6; ***P < 0.0001). *F*, effect of picrotoxin (20 μm), a GlyR channel blocker, on steady‐state EC_50_ glycine‐activated currents for α1, α1β, α1^N46K^ and α1^N46K^β GlyRs (*n* = 4–6; ***P < 0.0001).

GlyRs can be modulated by several allosteric ligands (Lynch, 2004). Notably, Zn^2+^ binds to two discrete sites on GlyRs (Miller *et al*. 2005 *b*), causing potentiation and inhibition at low and high micromolar concentrations, respectively (Bloomenthal *et al*. 1994; Harvey *et al*. 1999; Laube *et al*. 2000; Nevin *et al*. 2003; Miller *et al*. 2005 *a*). Co‐ or pre‐applying Zn^2+^ with a glycine EC_20_ revealed that N46K had no effect on Zn^2+^ potentiation of the glycine current (Fig. 5 *A* and *B*), which was also unaffected by A52S (not shown). However, glycine‐activated currents were differentially inhibited by higher Zn^2+^ concentrations (100 μm), with homomeric WT receptors showing greater inhibition (80 ± 5% inhibition) compared to the N46K mutant (25 ± 11 %; *P* < 0.05; Fig. 5 *B*). By contrast, A52S presented a similar Zn^2+^ inhibition profile to WT (86 ± 1.6%; *n* = 5). Interestingly, another hyperekplexia mutation GlyRα1^E103K^ located in loop A (Chung *et al*. 2010), in a similar manner to N46K, reduced glycine potency (EC_50_ 910 ± 140 μm; *n* = 7) and abolished 100 μm Zn^2+^ inhibition without affecting Zn^2+^potentiation (data not shown).

Neurosteroids are endogenous molecules in the brain that are capable of modulating GlyRs depending on the receptor subunit composition (Belelli *et al*. 1999; Lynch, 2004; Fodor *et al*. 2006; Jin *et al*. 2009). Inhibition of a glycine EC_80_ response by pregnenolone sulphate (PS; 30 μm) was unaffected by either the N46K or A52S mutations (Fig. 5 *C*). However, potentiation by 10 μm tetrahydro‐deoxycorticosterone (THDOC) of glycine EC_20_ (189 ± 20%; Fig. 5 *D*; *n* = 11) and glycine EC_50_ (161 ± 19%; *n* = 5) responses for GlyR α1 were significantly reduced by N46K (EC_20_: 139 ± 9%, Fig. 5 *D*, *n* = 8, EC_50_: 123 ± 11%; *n* = 7). However, potentiation of the glycine EC_20_ (168 ± 16 %, *n* = 6) and EC_50_ (134 ± 10 %, *n* = 5) responses induced by 10 μm THDOC at the α1^A52S^ mutant was similar to WT. By comparison, GlyRs containing the β subunit were relatively insensitive to THDOC (Fig. 5 *D*).

### Picrotoxin sensitivity is reduced by N46K

Picrotoxin (PTX) inhibits homomeric GlyRs by blocking the ion channel, but heteromers, containing the β subunit, are relatively less sensitive (Pribilla *et al*. 1992; Handford *et al*. 1996; Fig. 5 *E* and *F*). Picrotoxin (20 μm) inhibited the EC_50_ peak glycine current for WT GlyRs by 61 ± 4 %, and for α1^A52S^ mutant GlyR by 46 ± 3 % (*n* = 5). By comparison, it caused less inhibition of equivalent glycine currents at GlyR α1^N46K^ (by 34 ± 6 %; Fig. 5 *E*; *P* < 0.05). The inhibition by 20 μm PTX of steady‐state glycine‐activated currents was also greater for WT (81 ± 3 %) and A52S receptors (80 ± 4 %; *n* = 5) compared to N46K (63 ± 7 %; Fig. 5 *F*; *P* < 0.05).

### Impact of N46K on single‐channel currents and agonist concentration jumps

**Figure 6:**
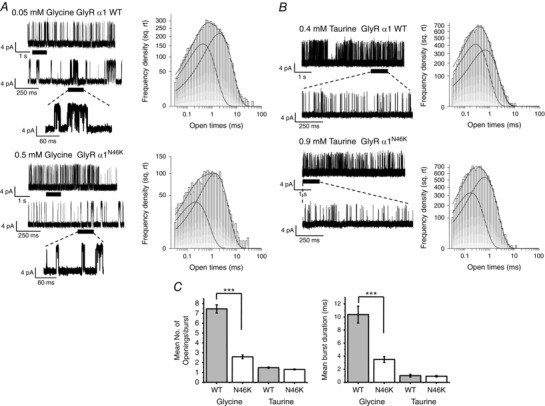
**Glycine and taurine single‐channel currents for WT and N46K GlyRs** *A*, left, cell‐attached recordings of single‐channel currents evoked by EC_60_ concentrations of glycine on HEK cells expressing homomeric WT and N46K GlyRs. Right, dwell time distributions for open states. Individual exponential density functions required to fit components of the open time distributions are shown, including the overall fit. *B*, left, cell‐attached recordings of single‐channel currents evoked by EC_60_ concentrations of taurine on WT and N46K GlyRs. Right, open time distributions for taurine currents fitted with exponential density functions. *C*, the mean number of openings per burst (left panel) and mean burst durations (right panel) were determined for WT and N46K in the presence of glycine and taurine. See Table 3 for values (***P < 0.0001).

To gain insight into how N46K affected the activation of GlyRs, single‐channel recording from cell‐attached patches was used. Single‐channel currents were activated by EC_60_ concentrations of glycine included in the patch pipette solution (Fig. 6 *A*). Using a patch potential of +100 mV, a comparison between wild‐type GlyRs and N46K mutant revealed a small but significant (*P* = 0.017) increase in channel current (WT, 6.35 ± 0.27 pA and N46K, 7.95 ± 0.37 pA; *n* = 4–5), which overall equates to a unitary conductance range of 65–80 pS. However, since we are not controlling the HEK cell membrane potential in cell‐attached recording mode, this small difference in single channel current could arise from small changes to the membrane potential and thus the driving force.

The channel open time distributions for WT and N46K α1GlyRs were best described by two exponentials with time constants τ_O1_ and τ_O2_ and areas *A*_O1_ and *A*_O2_ (Table 3). However, in a manner reminiscent of the hyperekplexia mutant K276E (Lewis *et al*. 1998), N46K displayed significant differences in the burst lengths and number of openings per burst for glycine compared with WT (Fig. 6 *A* (expanded traces) and *C* and Table 3). The mean burst duration was ∼3 times longer with ∼3 times as many openings per burst for WT compared to N46K (Table 3; *P* < 0.05).

**Table 3:** GlyRα1 single‐channel parameters

**Agonist**	**Glycine**		**Taurine**	
**GlyR**	**WT**	**N46K**	**WT**	**N46K**
Open time τ_1_ (ms)	0.34 ± 0.048	0.25 ± 0.036	0.30 ± 0.043	0.28 ± 0.048
Area 1 (%)	44.39 ± 1.46	33.38 ± 1.53*	53.38 ± 3.31	51.91 ± 6.54
Open time τ_2_ (ms)	1.37 ± 0.26	1.56 ± 0.23	0.78 ± 0.074	1.06 ± 0.13
Area 2 (%)	56.8 ± 0.82	68.48 ± 1.45*	52.07 ± 3.21	51.99 ± 6.94
Shut time τ_C1_ (ms)	0.12 ± 0.007	0.099 ± 0.012	0.11 ± 0.011	0.088 ± 0.003
Area C1 (%)	62.88 ± 8.65	47.44 ± 7.59	25.92 ± 2.63	20.62 ± 1.89
Shut time τ_C2_ (ms)	0.44 ± 0.034	0.59 ± 0.25	1.76 ± 0.58	6.36 ± 3.95
Area C2 (%)	31.15 ± 4.62	16.36 ± 5.17	13.96 ± 4.23	19.14 ± 4.91
Mean open time (ms)	1.22 ± 0.188	1.2 ± 0.152	0.843 ± 0.234	0.674 ± 0.054
Mean burst duration (ms)	10.4 ± 1.3	3.5 ± 0.46*	1.02 ± 0.21	0.93 ± 0.11
Mean number of openings per burst	7.5 ± 0.4	2.6 ± 0.2*	1.5 ± 0.06	1.3 ± 0.04
Mean closed time within burst (ms)	0.296 ± 0.042	0.25 ± 0.034	0.215 ± 0.0291	0.197 ± 0.026

Exponential open (τ_O_) and shut time (τ_C_) constants and their associated areas. Numbers of bursts, and burst lengths are shown for single‐channel currents activated by GlyR agonists: glycine and taurine, for WT and N46K GlyRα1. Only time constants for the two briefest shut states are shown to ensure shut times are measured bursts. All values are means ± SEM (*n* = 4–6; *Significant difference from the WT value; *P* < 0.05 for 6000–12,000 transitions per patch).

Single‐channel currents activated by EC_60_ concentrations of the partial agonist taurine were also investigated. Taurine‐activated channel current amplitudes were similar between WT and N46K GlyRs (7.63 ± 0.4 pA (WT) and 7.37 ± 0.5 pA (N46K); *n* = 5–6). Furthermore, the taurine open time distributions were very similar for WT and N46K GlyRs (Fig. 6 *B*; Table 3), and unlike glycine, the number of openings per burst and mean burst durations evoked by taurine were not notably different between WT and N46K GlyRs (Fig 6 *B* and *C*; Table 3).

**Figure 7:**
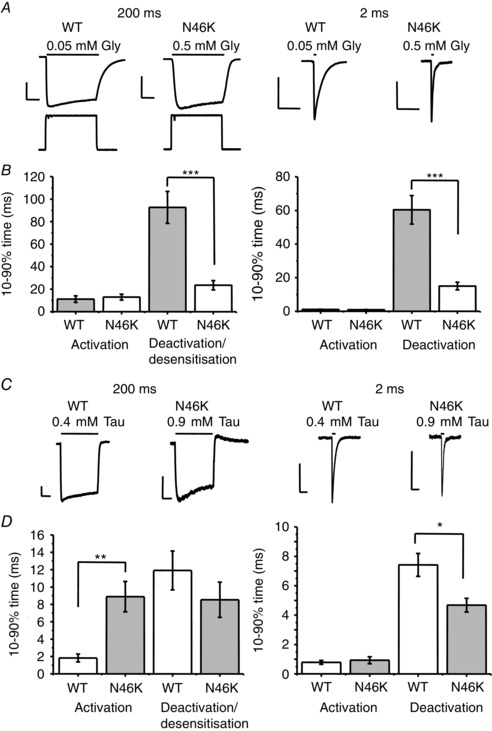
**Glycine currents deactivate faster for N46K compared to WT GlyRs** *A*, glycine currents recorded from outside‐out macropatches evoked by applying EC_60_ concentrations of glycine (Gly) for either 200 ms (left panel) or 2 ms (right panel) on HEK cells expressing WT GlyRs and N46K GlyRs. Open tip responses are shown in response to a pulse of 50% physiological salt solution/H_2_O. Calibration bars are 50 ms and 200 pA. *B*, bar graphs quantify the 10–90% glycine activation and deactivation/desensitisation rates (*n* = 6–9; ***P < 0.0001). *C*, recordings from outside‐out macropatches evoked by EC_60_ concentrations of taurine (Tau) for 200 ms (left panel) or 2 ms (right panel) on HEK cells expressing WT GlyRs (0.4 mm) and N46K GlyRs (0.9 mm). Calibration bars are 50 ms and 50 pA. *D*, bar graphs report the taurine 10–90% activation rates and also the deactivation/ desensitisation rates (*n* = 6–9; *P < 0.05; **P < 0.005).

The changes detected for burst durations of glycine‐activated channels were investigated further by using a fast application system to apply concentration jumps of glycine or taurine to outside‐out macropatches containing either WT or N46K GlyRs (Fig 7). A 200 ms application of EC_60_ glycine revealed a significantly faster 10–90% deactivation/desensitisation time for the N46K mutant (26.1 ± 4.4 ms; *n* = 9) compared with WT (98.2 ± 10.9 ms; *P* < 0.05; *n* = 10) with no change in the activation kinetics (Fig. 7 *A* and *B*).

Similarly, 2 ms applications of equipotent glycine concentrations also revealed significantly faster current deactivation for N46K (15.0 ± 2.3 ms; *n* = 6) compared with WT receptors (60.4 ± 8.5 ms; *P* < 0.05; *n* = 6) without affecting activation kinetics (WT; 1.10 ± 0.14 ms and N46K; 0.92 ± 0.09 ms; Fig. 7 *A* and *B*). Fast 200 ms application of EC_60_ taurine presented a different profile to glycine, with N46K exhibiting slower activation kinetics with no apparent change in deactivation/desensitisation kinetics. However, the 2 ms taurine pulse showed faster deactivation with N46K compared to WT (Fig. 7 *C* and *D*).

Overall, these data suggest that N46, located in close proximity to the GlyR binding loops A and D, is important for determining the duration of receptor activation by glycine, predominantly by regulating the deactivation rate.

## Discussion

### Molecular mechanisms underlying startle disease

Dysfunctional glycinergic neurotransmission is the major cause of human startle disease, with GlyRα1 gene mutations being the predominant cause. Various mechanisms have been proposed to account for the pathogenicity of GlyR missense mutations, including: dysfunctional ligand binding and channel activation for dominant mutations, to aberrant receptor trafficking and assembly for recessive mutations (Davies *et al*. 2010). However, we do not know how hyperekplexia mutations at subunit–subunit interfaces disrupt GlyR function. Do they displace the interfacial alignment between subunits – a necessary pre‐requisite for receptor activation – or do they hinder agonist access towards, and its retention at, the ligand binding site? The location of the N46K mutation provided an opportunity to address these questions whilst allowing insight into the activation mechanism for GlyRs and the phenotypic variability between *Glra1* hyperekplexia mouse mutants.

### Glycine receptor function and N46K

The *Nmf11* missense mutation in *Glra1*, resulting in a N46K substitution, exhibits recessive inheritance with a phenotype including small body size, handling‐induced tremor, intense whole‐body seizures and stiffness, an impaired righting reflex, and compromised survival by P21. However, transcription of *Glra1* and GlyR trafficking are unaffected by N46K as α1^N46K^β GlyRs still cluster at inhibitory synapses (Traka *et al*. 2006). Nevertheless, the *Nmf11* phenotype has all the hallmarks of severely compromised GlyR function normally associated with the loss‐of‐function, *oscillator* and *cincinnati* mutants.

The 9‐fold increase in the glycine EC_50_ caused by N46K would significantly reduce glycinergic inhibition, whilst the *spasmodic* mutation (A52S) in GlyRα1 (Lane *et al*. 1987; Ryan *et al*. 1994; Plested *et al*. 2007) increased glycine EC_50_ by only 2.5‐fold, possibly explaining why *spasmodic* shows exaggerated startle responses yet remains viable.

The glycine receptor is predominantly expressed as an α2 homomer in embryonic and early postnatal periods with a switch to α1β heteromers developing over time such that the heteromer becomes the dominant receptor population by P21 (Lynch, 2009). Both the α1^N46K^ and α1^N46K^β GlyRs exhibited reductions in glycine sensitivity compared with the WT equivalents and it is conceivable that the switch from α2 to α1^N46K^β GlyRs precipitates the phenotype and premature death at P21. In addition to the implications of increasing postsynaptic levels of α1^N46K^ GlyRs, causing dysfunction to glycinergic transmission, the identification of presynaptic α1 homomeric GlyRs (Turecek & Trussell, 2001) could also contribute to the disease phenotype (Xiong *et al*. 2014). Presynaptic GlyRs are thought to promote glycine release by depolarising axon terminals due to Cl^˗^ efflux (Turecek & Trussell, 2001; Jeong *et al*. 2003). We would expect presynaptic α1^N46K^ GlyRs to impair such a depolarisation, reducing glycine release, essentially resulting in disinhibition. This effect will further exacerbate the dysfunction to glycinergic transmission leading to hyperekplexia.

Given the developmental profile of α1β heteromers at P20 glycinergic synapses, we would expect glycine to act as an inhibitory neurotransmitter such that compromising receptor activity with the N46K mutation should exacerbate neural circuit excitation. At earlier times (P0–2), glycine fulfils an excitatory role as a consequence of high internal Cl^˗^ levels in neurons. However, it is doubtful that the N46K mutation would be effective during this earlier period given the relative paucity of α1β receptors at this stage of development.

Overall, the correlation between phenotype severity and extent to which GlyR sensitivity to glycine is reduced, under conditions where receptor trafficking and maximal glycine currents are unaffected, may be an important criterion for future genotype‐phenotype studies of human hyperekplexia.

### N46K is unlikely to affect ion channel gating

The unaltered maximal glycine‐activated current for GlyR α1^N46K^ suggested that gating efficiency was possibly unaffected. However, if agonist efficacy (*E*) is very high, then a reduction in *E* would appear to displace the curve with minimal reduction in the relative maximum response. By using estimates of *E* for glycine activating the fully liganded GlyR α1 (∼13–20; Lewis *et al*. 2003; Lape *et al*. 2008), coupled to appropriate values for agonist dissociation constants and a simple linear kinetic model, a 9‐fold shift in the glycine curve by reducing *E* alone would cause the maximum response to fall by ∼60%. Similarly, N46K caused 6‐fold and 3‐fold shifts in the β‐alanine and taurine curves, but it did not reduce the maximal response when compared with glycine. From our receptor model, (β‐alanine *E* = 9 (Lewis *et al*. 2003); taurine *E* = 3 (Lewis *et al*. 1998)), we would have expected readily observable 55% to over 70% reductions in the maximum responses, respectively, if channel gating was affected. Similar considerations of the pre‐activation state (Lape *et al*. 2008), which can be used to distinguish full from partial agonists (Lape *et al*. 2008; Miller & Smart, 2010), also suggested that reduced formation of such a state is unlikely to account for the N46K phenotype, though kinetic modelling (see below) indicated a potential effect of N46K on one, triply liganded pre‐activation state.

### Effect of N46K on partial agonist and competitive antagonist binding

Compared with the full agonist glycine, β‐alanine, taurine and GABA have lower affinities for and efficacies at WT GlyRs, exhibiting depressed, right‐shifted curves. However, GlyRα1^N46K^ did not depress these curves further, and the shifts were notably smaller for the weaker agonists. GABA and taurine may have different binding profiles compared to glycine, potentially involving residues that may be unimportant for glycine, and thus conceivably less affected by N46K.

The prospect that N46K reduces glycine binding is reinforced by the reduction in strychnine inhibition, an effect that does not occur with A52S. Although strychnine and glycine most likely bind to overlapping sites on GlyRs (Grudzinska *et al*. 2005; Brams *et al*. 2011), our receptor models indicate the lysine side‐chain is too short to directly inhibit glycine binding, but could hinder (by charge and/or volume) the binding of the much larger strychnine molecule. N46K induced a comparable 15‐fold shift in the strychnine inhibition curve compared with the 9‐fold shift for glycine.

### N46 stabilises the receptor binding site

The importance of the amide side‐chain at position 46 for maintaining the glycine sensitivity of WT GlyRs was evident following substitution with residues that have bulky hydrophobic (Trp, Phe) or charged (Lys, Arg, Glu, Asp) side‐chains. These all caused large displacements to the glycine curve that did not occur with N46Q, which retains the amide moiety. The location of N46 near the subunit interface and its apparent effect on glycine binding, suggested it might interact with residues either located on the adjacent α subunit or on β loops within the same α subunit that are important for ligand binding. GlyR structures suggest N46 points towards loop A, which is important for binding in nicotinic AChRs (Cashin *et al*. 2007), GABA_A_Rs (Padgett *et al*. 2007) and GlyRs (Miller *et al*. 2008), though N46 was unlikely to interact directly with E103 or N102 given their side‐chain orientation. However, loop A (β4 loop) could still be affected by N46K, particularly as Zn^2+^ binding residues are nearby on β5 loop and N46K reduced Zn^2+^ inhibition.

A parsimonious explanation for the N46 phenotype may involve important ligand binding residues, on loop D, which are upstream of N61 (Grudzinska *et al*. 2005). Homology modelling, and recent GlyR structures (Du *et al*. 2015; Huang *et al*. 2015) suggest N46 and N61 are juxtaposed (less than 3 Å apart) with the strychnine binding residue, R131 in loop E, and loop A, all in the same plane (Fig. 4 *A*). The charge reversal experiments involving N46 and N61 demonstrated that these two asparagines could potentially interact, particularly given the likelihood of de‐protonated carboxyl groups in their side‐chains under physiological conditions. Possible interactions between the two carboxamide side‐chains could include: electronic delocalisation, dipole–dipole or charge–charge interactions. If these interactions are disrupted, as indicated by the charged residue substitutions, this could alter the structural integrity of the binding site located just above this plane, thus reducing glycine binding.

### Allosteric modulators

In regard to allosteric modulation, potentiation by Zn^2+^ was unaffected by either N46K or A52S, but Zn^2+^ inhibition was reduced by N46K. Both H107 and H109 on β5 loop (just outside loop A) constitute the Zn^2+^ inhibition site (Harvey *et al*. 1999; Nevin *et al*. 2003; Miller *et al*. 2005 *a*). Since N46 faces loop A, across the subunit interface, substitution with a positively charged lysine could disrupt loop A, perturbing Zn^2+^ coordination and compromising inhibition. This is supported by the results with A52S, which is located further along the β strand and thus away from loop A, and did not disrupt Zn^2+^ inhibition when compared with the WT. Interestingly, E103K (loop A), a mutation that causes hyperekplexia in humans (Chung *et al*. 2010), also reduced Zn^2+^ inhibition. By comparison with Zn^2+^, neurosteroid potentiation at GlyRs was also reduced by N46K. This could contribute towards lethality considering that the non‐lethal mutation, A52S, exhibited a similar sensitivity to THDOC compared to WT. The inhibition produced by picrotoxin, a GlyR channel blocker, was also reduced by N46K, indicating that ‘longer‐range’ structural effects can result from this mutation, which was less evident with the A52S mutation.

### Effect of N46K on single‐channel currents and fast concentration jumps

Single‐channel data provided insight into how N46K may reduce the potency of glycine. The major effect appeared to involve reductions in the mean burst duration and mean number of openings per burst compared with WT GlyRs. By contrast, the same parameters were seemingly unaffected by taurine, reflecting the smaller shift in the concentration curves by N46K. Using glycine concentration jumps identified a significantly faster deactivation of GlyRα1^N46K^ compared to WT, suggesting that N46K may destabilise the orthosteric site allowing glycine to dissociate faster. Generally, the deactivation kinetics for taurine were much faster than for glycine and the differences between WT and N46K were similar but less prominent. By comparison to other hyperekplexia‐inducing mutations, A52S caused a reduction in the co‐operativity between glycine binding sites without affecting gating (Plested *et al*. 2007), whilst the hyperekplexia mutation K276E hinders channel opening causing shorter mean open times and reduced whole‐cell currents without affecting gating (Lewis *et al*. 1998).

**Figure 8:**
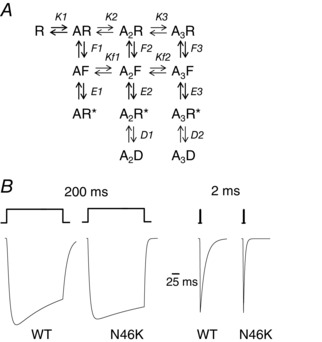
**Simulations of glycine currents at WT and N46K GlyRα1** *A*, a kinetic model of the GlyR depicting 4 shut states (R, AR, A_2_R and A_3_R), with 3 of these bound with up to 3 molecules of glycine (*A*). Once agonist is bound, the AR, A_2_R and A_3_R states can undergo pre‐activation conformational transitions to states AF, A_2_F and A_3_F, which are still shut states. These states can then undergo a gating reaction to form AR*, A2R* and A3R*, which are open conducting states. Two of these, A_2_R* and A3R* can enter into agonist‐bound desensitised states (A_2_D and A_3_D) when exposed to higher agonist concentrations. Here, *K* is the agonist dissociation constant (unbinding/binding rate = *k* ˗ 1/*k*1) taking account of statistical factors for agonist binding and unbinding; *Kf* is the agonist dissociation constant (*k*_f‐_/*k*_f+_) for the pre‐activation states; *F* is the pre‐activation conformation constant, = *f*_1_/*f*_‐1_; *E* is the gating constant, (= β/α); and *D* represents the desensitisation constant (= δ_1_/δ_‐1_; *F*, *E* and *D* = forward/backward rates). *B*, predicted matched glycine‐activated currents for WT (left, 50 μm) and N46K GlyRs (right, 500 μm) using the model described in *A*. Glycine was applied for either 2 or 200 ms. Note the faster deactivations for N46K which largely result from increases in *K* and particularly in *Kf* for the transition, A_2_F ↔ A_3_F. See text for details.

To account for the effects caused by N46K, we constructed a three binding site model for the GlyR that incorporated pre‐activation states (Burzomato *et al*. 2004) and was modified to include two desensitisation states (Fig. 8 *A*). To describe the changes to the glycine current profiles and agonist concentration–response curves by N46K required several empirical changes to rates and constants. Primarily, the deactivation rates (and thus the agonist dissociation constants (*K*1–3)) between states AR, A2R and A3R were increased (∼5‐fold) together with a reduction in the pre‐activation constant, *F*3 (from A_3_R to A_3_F, ∼9‐fold; Table 4) to displace the glycine concentration–response curve. To define the change in the deactivation kinetics, the enhanced decay rates observed for N46K were largely accounted for by increasing the agonist unbinding rate (and thus *K*_f2_) from the pre‐activation state A_3_F to A_2_F by ∼5‐fold (Fig. 8; Table 4). There are some similarities here with the changes induced by A52S (Plested *et al*. 2007) including the reduction in *F*3 (9‐fold) and increase in *K* (∼3‐fold), which may reflect, given the relative proximity of N46 and A52 in terms of primary sequence, the impact this part of the extracellular domain has on GlyR function.

**Table 4:** Parameters used to generate the simulated currents in Fig. 8

**Rate constants**	**WT**	**N46K**
*K*1	150 μm	727 μm
*K*2	120 μm	615 μm
*K*3	9.4 μm	47 μm
*F*1	0.73	0.6
*F*2	6	6.6
*F*3	23.4	2.99
*Kf*1	22 μm	56 μm
*Kf*2	22 μm	104 μm
*E*1	1.45	0.73
*E*2	14.75	9
*E*3	23.3	35
*D*1	5.7	8.5
*D*2	1.1	1.1

**Figure 9:**
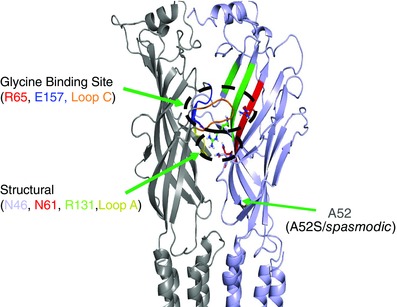
**Structural model of the GlyR** Model showing a side‐view of the GlyRα1 subunit interface highlighting the important structural residues, N46, N61 and R131, all residing in the same plane as loop A and positioned below the orthosteric glycine binding site, involving residues: R65, E157 and loop C.

In conclusion, the N46K missense mutation markedly reduced glycine sensitivity, resulting in a severe, lethal startle phenotype. N46 most likely interacts with N61 to stabilise binding loops D and E near the orthosteric site for glycine. By disrupting the structural integrity of the glycine site (Fig. 9) N46K promotes agonist unbinding from the orthosteric site, particularly from the triple agonist molecule bound pre‐activation state, to cause faster GlyR deactivation. This mechanism reveals a novel pathogenic effect for a hyperekplexia‐inducing mutation.

## Additional information

### Competing interests

The authors have no competing interests to declare.

### Author contributions

The work for this study was carried out at University College London. M.E.W. designed and performed the experiments, acquired, analysed and interpreted the data, and helped write the manuscript; A.C. contributed data and performed data analysis; M.C.G. contributed towards the homology modelling and interpreted data; R.J.H. contributed to the conception of the work and to manuscript writing; T.G.S. conceived and designed the study, performed kinetic modelling and simulations, and helped to write the manuscript. All authors have approved the final version of the manuscript and agree to be accountable for all aspects of the work. All persons designated as authors qualify for authorship, and all those who qualify for authorship are listed.

### Funding

This work was supported by the MRC, EU‐FP7 consortium, Neurocypres, and The Leverhulme Trust.
